# One fold, many functions—M23 family of peptidoglycan hydrolases

**DOI:** 10.3389/fmicb.2022.1036964

**Published:** 2022-10-21

**Authors:** Alicja Razew, Jan-Niklas Schwarz, Paweł Mitkowski, Izabela Sabala, Magdalena Kaus-Drobek

**Affiliations:** Laboratory of Protein Engineering, Mossakowski Medical Research Institute, Polish Academy of Sciences, Warsaw, Poland

**Keywords:** peptidoglycan hydrolases, M23 peptidases, enzybiotics, *Staphylococcus aureus*, drug-resistance, lysostaphin

## Abstract

Bacterial cell walls are the guards of cell integrity. They are composed of peptidoglycan that provides rigidity to sustain internal turgor and ensures isolation from the external environment. In addition, they harbor the enzymatic machinery to secure cell wall modulations needed throughout the bacterial lifespan. The main players in this process are peptidoglycan hydrolases, a large group of enzymes with diverse specificities and different mechanisms of action. They are commonly, but not exclusively, found in prokaryotes. Although in most cases, these enzymes share the same molecular function, namely peptidoglycan hydrolysis, they are leveraged to perform a variety of physiological roles. A well-investigated family of peptidoglycan hydrolases is M23 peptidases, which display a very conserved fold, but their spectrum of lytic action is broad and includes both Gram- positive and Gram- negative bacteria. In this review, we summarize the structural, biochemical, and functional studies concerning the M23 family of peptidases based on literature and complement this knowledge by performing large-scale analyses of available protein sequences. This review has led us to gain new insight into the role of surface charge in the activity of this group of enzymes. We present relevant conclusions drawn from the analysis of available structures and indicate the main structural features that play a crucial role in specificity determination and mechanisms of latency. Our work systematizes the knowledge of the M23 family enzymes in the context of their unique antimicrobial potential against drug-resistant pathogens and presents possibilities to modulate and engineer their features to develop perfect antibacterial weapons.

## Introduction

Peptidases are found in all living organisms. This large family of enzymes catalyzes the hydrolytic disintegration of peptide bonds in proteins or peptides. Their genes are broadly disseminated across a tree of life. They account for almost 6% of the total human proteome, whereas in bacteria, this proportion is approximately 3% (2.85% of *Escherichia coli* proteins and up to 3.99% of *Bacillus cereus*; Barrett, 2004; Potempa and Pike, 2004). The majority of bacterial peptidases (almost 90%) are serine, metallo- and cysteine proteases, whereas aspartic and threonine peptidases contribute less than 10% to the total (Page and Di Cera, 2008). Bacterial peptidases are leveraged by bacteria to fulfil a multitude of biological roles related to cell physiology, replication, survival, and virulence.

A large group of bacterial proteolytic enzymes acts as peptidoglycan hydrolases (PGHs), which is a diverse group of enzymes of different folds and specificities. A common theme in their function is their ability to digest peptidoglycan (PG), a polymer forming a scaffold of the bacterial cell wall. PGHs cleave nearly every bond in PG; generally, multiple enzymes target the same PG bond (Firczuk and Bochtler, 2007). PGHs are engaged in PG maturation, turnover, and recycling during growth and division (Vollmer et al., 2008). Apart from being involved in cell wall metabolism, PGHs also act as bacteriocins that eliminate bacterial competitors residing in the same ecological niche as their bacterial host. Due to their prominent lytic activity against bacteria, including antibiotic-resistant strains, PGHs are regarded as a promising alternative to conventional antimicrobials, therefore, they are much needed in the times of rapid spread of drug-resistant bacteria (Terreni et al., 2021).

The metallopeptidases are structurally the most diverse group of PGHs, therefore, several families have been described based on their overall fold (Firczuk and Bochtler, 2007). Among them are the LytM-type enzymes that are also classified as the peptidase family M23 (MEROPS database; Rawlings et al., 2008). Over the years, many M23 peptidases have been characterized structurally, biochemically, and functionally (Table 1). Their bactericidal potential against pathogenic *Staphylococcus aureus* has been explored, where they reached the stage of clinical trials, including a holotype of the M23 family – the lysostaphin. Here, we present a current state of knowledge on M23 peptidases with a special focus on PG hydrolytic enzymes. In addition to data from previous literature, we analyzed a large set of M23 deposits, especially their domain architecture and net charge, to complement missing information on this common family of PGHs.

**Table 1:** A list of characterized M23 peptidases, their sources, specificities, and biological roles.

Protein	Original organism	M23 catalytic domain activity	Specificity	Role	PDB ID	References
Lysostaphin (Lss)	*Staphylococcus simulans* biovar *staphylolyticus*	Glycyl-glycine endopeptidase	Gly-Gly	Bacteriocin	4QPB 4LXC	Schindler and Schuhardt, 1964; Simmonds et al., 1996; Bastos et al., 2010; Sabala et al., 2014; Tossavainen et al., 2018
LytM	*Staphylococcus aureus*	Glycyl-glycine endopeptidase	Gly-Gly	Autolysin	1QWY 2B44 2B13 2B0P 4ZYB	Odintsov et al., 2004; Firczuk et al., 2005; Grabowska et al., 2015
LytU	*Staphylococcus aureus*	Glycyl-glycine endopeptidase	Gly-Gly	Bacteriocin/Peptidoglycan remodeling	5KQB 5KQC	Raulinaitis et al., 2017
ALE-1	*Staphylococcus capitis* EPK1	Glycyl-glycine endopeptidase	Gly-Gly	Bacteriocin	-	Fujiwara et al., 2005
SpM23_A	*Staphylococcus pettenkoferi*	Glycyl-glycine endopeptidase	Gly-Gly	Autolysin	-	Wysocka et al., 2021
SpM23_B	*Staphylococcus pettenkoferi*	Glycyl-glycine endopeptidase	Gly-Gly	Bacteriocin	-	Wysocka et al., 2021
gp13	*Bacillus subtilis* phage ф29	D,D-endopeptidase	D-Ala-Dpm	Phage tail-associated peptidoglycan hydrolase	3CSQ	Xiang et al., 2008
Csd2	*Helicobacter pylori*	D,D-endopeptidase	D-Ala-Dpm	Cell shape determinant	5J1K 5J1M	An et al., 2016
ShyA	*Vibrio cholerae*	D,D-endopeptidase	D-Ala-Dpm	Growth promoting endopeptidase	2GU1 6U2A 6UE4	Shin et al., 2020
YebA	*Escherichia coli*	D,D-endopeptidase	D-Ala-Dpm	Peptidoglycan turnover	-	Singh et al., 2012
NMB0315	*Neisseria meniningitids*	D,D-endopeptidase	D-Ala-Dpm	Bacterial virulence	3SLU	Wang et al., 2011
CwlP	*Bacillus subtills* SP-β-prophage	D,D-endopeptidase	D-Ala-Dpm	Phage encoded endolysin		Sudiarta et al., 2010
Csd3 (HdpA)	*Helicobacter pylori*	D,D-endopeptidase D,D-carboxypeptidase	D-Ala-Dpm D-Ala- D-Ala	Cell shape determinant	4RNY 4RNZ	An et al., 2015
Pgp3	*Campylobacter jejuni*	D,D-endopeptidase D,D-carboxypeptidase	D-Ala- Dpm D-Ala- D-Ala	Peptidoglycan reshaping	6JMX 6JM 6KV1 6JN0 6JN1 6JMZ 6JN7 6JN8	Min et al., 2020
LytH (YunA)	*Bacillus subtilis*	L,D-endopeptidase	L-Ala-D-Glu	Spore cortex formation		Horsburgh et al., 2003
Enterolysin A	*Enterococcus faecalis* B9510	L,D-endopeptidase	L-Ala-D-Glu; L-Lys-D-Asp	Bacteriocin	-	Nilsen et al., 2003; Khan et al., 2013
2638A endolysin	*Staphylococcus aureus* phage 2638A	D,L- endopeptidase	D-Ala-Gly	Phage encoded endolysin	6YJ1	Abaev et al., 2013; Eichenseher et al., 2022
EnpA	*Enterococcus faecalis* phage 03	D,L-endopeptidase	D-Ala- Gly/Ala/Ser	Phage encoded endolysin	6SMK	Reste de Roca et al., 2010; Małecki et al., 2021
Zoocin A	*Streptococcus equi* subsp. *zooepidemicus* 4,881	D,L-endopeptidase	D-Ala-L-Ala	Bacteriocin	5KVP	Simmonds et al., 1996; Akesson et al., 2007; Xing et al., 2017
LasA	*Pseudomonas aeruginosa*	Endopeptidase	Gly -Gly/Tyr/Leu/Phe	Bacterial virulence	3IT5 3IT7	Grande et al., 2007; Spencer et al., 2010
Pseudoalterin	*Pseudoalteromonas sp.* strain CF6-2	Endopeptidase	Gly-Gly/Ala D-Ala-D-Ala D-Ala-L-Lys L-Lys-D-Ala Desmosine-Ala-Ala Desmosine-Ala-Ala-Ala	Elastin degradation in marine vertebrates	6IK4	Zhao et al., 2012; Tang et al., 2020
Fibronectin binding proteins (e.g., TDE1738)	*Trepanoma denticola*	Endopeptidase	Unknown	Fibronectin-binding protein	-	Bamford et al., 2010
Tal2009	*Lactococcus lactis subsp. cremoris* UC509 phage Tuc2009	Endopeptidase	Unknown	Phage tail-associated lysin		Kenny et al., 2004
Atu4178	*Agrobacterium tumefaciens*	Endopeptidase	Unknown	Polar growth		Figueroa-Cuilan et al., 2021
EnvC	*Escherichia coli*	LytM factor	-	Septum-specific activation of amidase AmiA/B	-	Uehara et al., 2009; Peters et al., 2013
NlpD	*Escherichia coli*	LytM factor	-	Septum-specific activation of amidase AmiC	-	Uehara et al., 2009; Peters et al., 2013
DipM	*Caulobacter crescentus*	LytM factor	-	Peptidoglycan remodeling	-	Goley et al., 2010; Möll et al., 2010

## The substrate: Peptidoglycan (PG)

**Figure 1:**
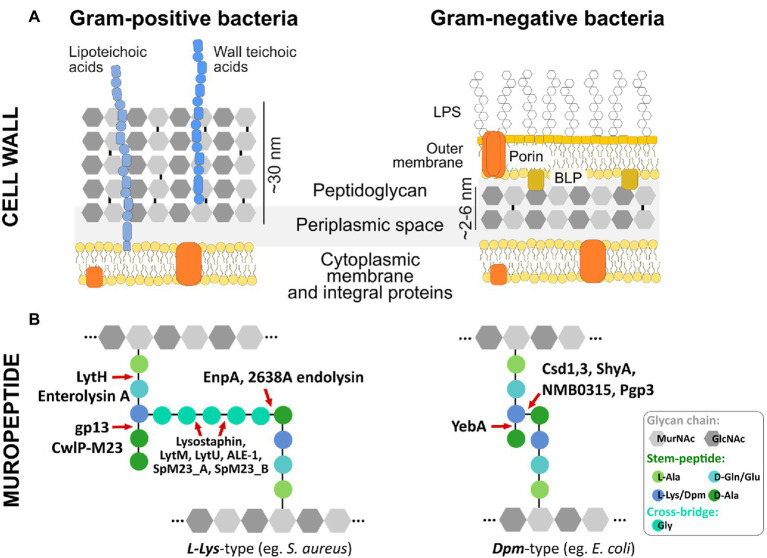
Bacterial cell wall architecture, peptidoglycan composition, and M23 hydrolytic enzymes dedicated to different bonds in PG structure **(A)** Schematic representation of Gram-positive and Gram-negative bacteria cell wall. **(B)** Basic building blocks and crosslinking of peptidoglycan layer depicted for representative species of Gram-positive (e.g., *S. aureus*) and Gram-negative (e.g., *E. coli*) bacteria. M23 peptidases are presented as enzymes dedicated to cleaving specific bonds in peptidoglycan structures of both *L-Lys*-type and *Dpm*-type peptidoglycan. Glycan chains of N-acetylmuramic acid (MurNAc) and N-acetylglucosamine (GlcNAc) are depicted as gray hexagons; integral membrane proteins are colored in orange; D- and L-amino acids and *Dpm* (*meso*-diaminopimelic acid) characteristic for the stem peptide and cross-bridge are presented as colored circles; LPS, lipopolysaccharides; BLP, Braun’s lipoprotein (Vollmer et al., 2008).

PG is a key component of the bacterial cell wall that helps maintain cell shape and prevents bacteria from lysis through osmotic rupture (Höltje, 1998). In addition to its mechanistic role in keeping bacterial rigidity and integrity, the PG layer provides a certain degree of flexibility to allow bacteria to move, grow, and divide. In Gram-positive bacteria, PG is thick (ca. 30–50 nm) and multi-layered with covalently attached cell wall compounds, such as wall teichoic- and lipoteichoic acids. In contrast, Gram-negative bacteria contain a thin (ca. 1.5–15 nm) and predominantly single PG layer located in the periplasm surrounded by an inner and an outer LPS-rich membrane (Figure 1, panel A; Vollmer et al., 2008).

The basic building block of PG is a glycan chain made up of repeating N-acetylglucosamine (GlcNAc) and N-acetylmuramic acid (MurNAc) residues linked by β-1,4 bonds. A short peptide chain called stem peptide protrudes from the glycan chain (Figure 1, panel B). In most Gram-positive bacteria, stem peptide comprises L-Ala-D-iGlu-L-Lys-D-Ala-D-Ala, while in Gram-negatives the most common amino acid located at the third position is *Dpm* (*meso*-diaminopimelic acid) that directly links D-Ala to an adjacent stem peptide attached to a neighboring glycan strand (Figure 1, panel B). Therefore, depending on the third position in the stem peptide, the bacterial peptidoglycans are classified as *L-Lys*-type or *Dpm*-type (Firczuk and Bochtler, 2007). While the PG composition of Gram-negative bacteria is conserved, Gram-positive bacteria display huge diversity in terms of PG composition and structure, particularly in their cross-bridge that links adjacent stem peptides. These differences are observed mostly at the species level, but certain variabilities have been found among strains or even serovars (Vollmer et al., 2008). PG composition also differs depending on the form in which bacteria exist in the environment; PG isolated from bacteria residing in biofilms is different compared to PG derived from planktonic cells (Chang et al., 2018; Anderson et al., 2020). The differences include stem peptide composition and other modifications that come from the action of PG-modifying enzymes, including PGHs, deacetylases, and lytic transglycosylases (Anderson et al., 2020).

## The enzymes: Peptidoglycan hydrolases (PGHs)

There are more than 100 types of PG in bacteria that differ in their composition, architecture, length, and thickness (Schleifer and Kandler, 1972; Smith et al., 2000). To process such complicated structures, bacteria produce diverse PGHs. According to their catalytic mechanism, PGHs are grouped into amidases that cleave the bond between MurNAc and the first residue (L-Ala) of the stem peptide; glycosidases, such as muramidase that hydrolyze the β-(1,4)-glycosidic linkage between MurNAc and GlcNAc; and glucosaminidases that cleave the link between GlcNAc and MurNAc. Peptidases are further subdivided into carboxy- and endopeptidases that can cleave off the C-terminal amino acid or cut between the amino acids, respectively. Endopeptidases are further classified into LD-, DL-, or DD- endopeptidases depending on the enantiomers forming a scissile bond (Vermassen et al., 2019).

Based on their origin and function, PGHs can be grouped into autolysins, exolysins, and endolysins. Autolysins are endogenous lytic enzymes that break down the PG components, therefore, enabling cell separation following cell division (Jaenicke et al., 1987). LytA autolysin is an *N*-acetylmuramoyl-L-alanine amidase in *Streptococcus pneumoniae.* It is located in the cell envelope and is involved in a variety of physiological functions associated with cell wall growth, metabolism, and PG turnover (Whatmore and Dowson, 1999). Autolysins, AtlAEfm from *Enterococcus faecium* and AtlE from *Staphylococcus epidermidis*, are essential for extracellular DNA release into the biofilm matrix, therefore, contributing to biofilm stability and attachment to the surface (Qin et al., 2007; Paganelli et al., 2013). Exolysins (known as bacteriocins) represent a group of PGHs released by bacteria into the environment to act as weapons against other species that reside in the same ecological niche (Vermassen et al., 2019). The best-characterized example is lysostaphin (Lss), which was originally found *in Staphylococcus simulans* biovar *staphylolyticus*, where it eradicates *S. aureus* (Schindler and Schuhardt, 1964; Thumm and Götz, 1997; Bastos et al., 2010). Finally, endolysins are phage-encoded lysins that accumulate in the cytoplasm of phage-infected bacterial cells at the end of the lytic cycle. They can cleave peptidoglycan upon release to the cell wall through membrane lesions formed by holins. Such lysis enables progeny virions to be released (Young et al., 2000). Although autolysins, exolysins, and endolysins have different origins, they may have similar architecture and share the same specificity towards PG (Vermassen et al., 2019). In this review, they are grouped together under the term PGHs.

## M23 peptidases

**Figure 2:**
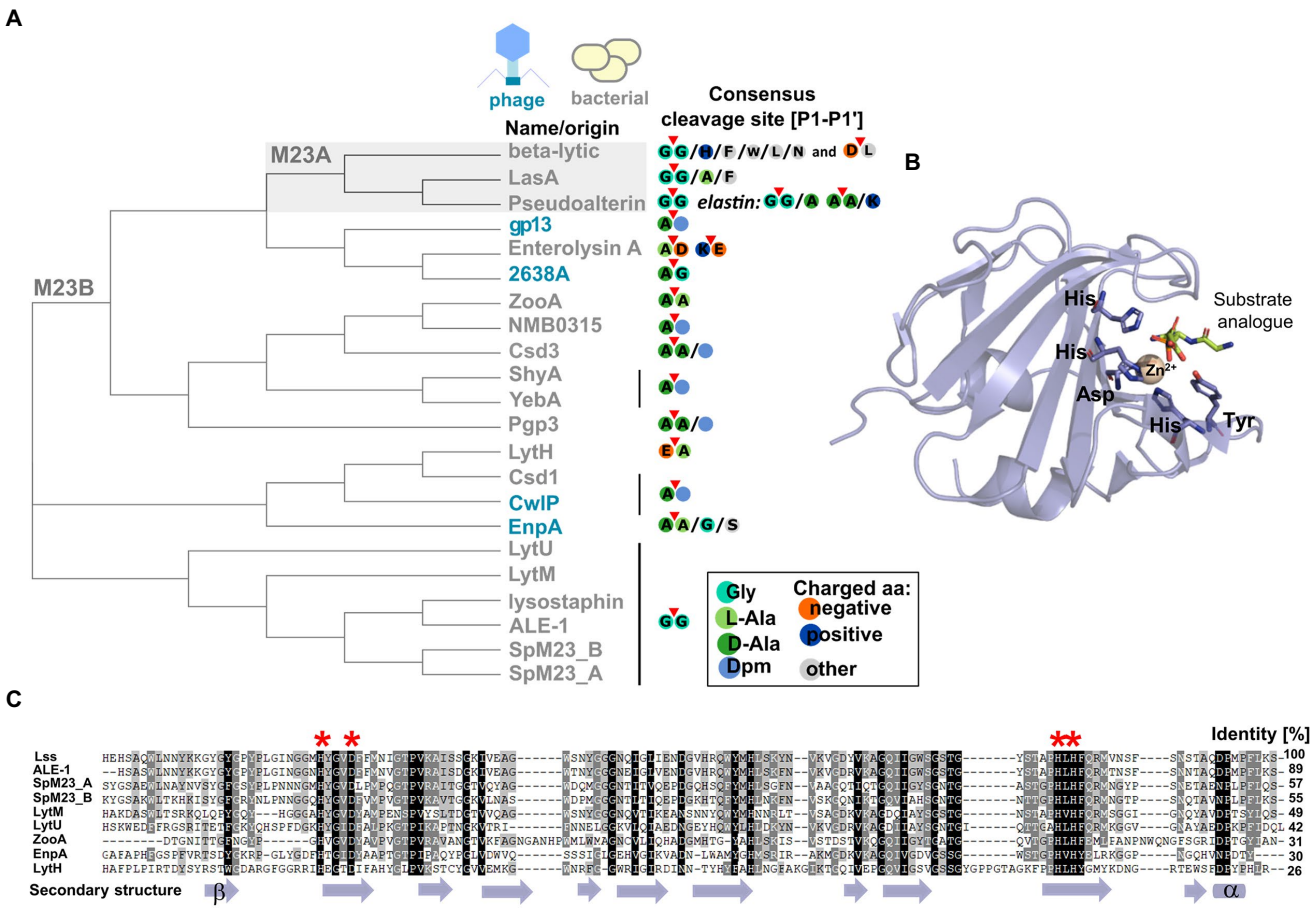
Characteristic features of M23 peptidases. **(A)** Phylogenetic analysis represented as a cladogram of M23 peptidases of phage (colored blue) or bacterial (colored gray) origin with their specificities. M23A subfamily was indicated against gray background. According to the nomenclature of protease substrate specificity defined by Schechter and Berger (Schechter and Berger, 1967), amino acid residues in the peptide substrate sequence are consecutively numbered outward from the cleavage sites as P4-P3-P2-**P1-P1’**-P2’-P3’-P4’ and the scissile bond is located between the **P1** and **P1’** positions (here marked with red triangle). **(B)** Overall fold of M23 peptidase domain represented by LytM catalytic domain (PDB ID: 4ZYB). The structure was solved in the presence of the transition state analogue and zinc ion coordinated by conserved motifs: Hx_3_D and HxH. **(C)** Multiple sequence alignment performed by ClustalX and presented as Gendoc of M23B representative proteins with highly conserved zinc-binding motifs (depicted in red asterisk on the top of alignment) and secondary structure elements (depicted in violet at the bottom) assigned by PROMALS3D program (Larkin et al., 2007; Pei et al., 2008).

The M23 peptidases are common PGHs, which are distinguished based on the conservation of two catalytic motifs: H(x)_n_D and HxH (Rawlings et al., 2008). The first discovered member of this family was beta-lytic protein BLP from *Lysobacter enzymogenes* (formerly *Achromobacter lyticus*) that lysed other bacteria and some soil nematodes (Whitaker, 1965). Due to sequence characteristics and broad sequence specificity, BLP was classified into the M23A subfamily together with staphylolysin (LasA) from *Pseudomonas aeruginosa* and pseudoalterin from *Pseudoalteromonas* sp. strain CF6-2. M23A members are particularly tolerant to sequence alterations at the P1’ position of the targeted bond (Figure 2, panel A).

The majority of known bacterial- and phage-encoded M23 peptidases belonged to M23B subfamily. Primary studies revealed that M23B displays a preference towards glycylglycine bond as has been demonstrated for Lss, which was originally found in *Staphylococcus simulans* biovar *staphylolyticus*, where it specifically disrupts pentaglycine cross-bridges of PG in the cell wall of *S. aureus* and its clinical and drug-resistant isolates (Schindler and Schuhardt, 1964; Thumm and Götz, 1997; Bastos et al., 2010; Kokai-Kun, 2012; Jayakumar et al., 2021). Other enzymes that target preferentially Gly (P1)-Gly (P1’) bonds are LytM, LytU (*S. aureus*), ALE-1 (*S. capitis*), SpM23_A, and SpM23_B (*Staphylococcus pettenkoferi*). However, certain M23B peptidases display distinct specificity, for instance, D,D-endopeptidases ShyA or YebA digest Gram-negative type PG (direct cross-bridge between Dpm (P1) and D-Ala (P1’)). In addition, D,L-endopeptidases zoocin A or EnpA show specificity toward a broader set of PG bonds, e.g., D-Ala (P1)-L-Ala (P1’) and D-Ala (P1)-L-Ala/Gly/L-Ser (P1’), respectively (Figure 1, panel B; Figure 2, panel A; Akesson et al., 2007; Reste de Roca et al., 2010; Singh et al., 2012; Shin et al., 2020).

All M23 enzymes are zinc-dependent metallopeptidases (Figure 2, panel B). A catalytic zinc ion is involved in the nucleophilic attack on the scissile bond. Certain M23 peptidases bind two zinc ions, which diminishes their lytic activity (LytU and Lss (Raulinaitis et al., 2017; Tossavainen et al., 2018)). For LytU, this mechanism was proposed to play a regulatory role over the enzyme activity that limits its activity to certain pH conditions (Raulinaitis et al., 2017). Several studies aiming at exchanging the zinc for other divalent ions (Co(II), Mn(II), and Cu(II)) proved successful, although with different outcomes depending on the enzyme. For instance, catalytic zinc exchange to Co(II) decreased the lytic activity of LytM and Lss by 20%, but increased the activity of LytU by 800% (Firczuk et al., 2005; Raulinaitis et al., 2017; Tossavainen et al., 2018).

### Sequence features

The majority of M23 peptidases contain both conserved motifs characteristic of the M23 family (Figure 2, panel C). However, there is a certain variation in the number of amino acids spacing the first catalytic residues, H(x)_n_D, that is observed not only between M23A and M23B subfamilies, but also within M23B alone. For example, catalytic residues of LasA (M23A) form a long, H(x)_12_D motif, whereas gp13 (M23B) displays an H(x)_7_D motif (Xiang et al., 2008; Spencer et al., 2010).

To get insights into variability within M23 peptidase sequences, we generated a neighbor-joining tree that reflects the relationships of the M23 peptidases defined in the Conserved Domains Algorithm (Sievers et al., 2011; Lu et al., 2020; Figure 2, panel A). Firstly, the analysis revealed that although M23A members cluster together, they do not form a distinct outgroup. Instead, they cluster with certain M23B peptidases, two phage enzymes (gp13 and 2638A), and enterolysin. Secondly, within the M23B subfamily, defined clusters overlap well with the defined substrate specificities. For instance, ZooA, NMB0315, Csd3, ShyA, YebA, and Pgp3 form a cluster of enzymes that preferentially target bonds between D-Ala at the P1 site and D-Ala or Dpm at the P1’ site. In addition, all glycyl-glycine endopeptidases cluster out. This finding demonstrates that sequence relatedness serves as a hallmark for a substrate preference, and discrepancies can be distinguished even within an enzyme family containing members with the same catalytic motifs. Lastly, the cladogram demonstrates that autolytic LytU and LytM are more distantly related than bacteriocins, lysostaphin, ALE-1, and SpM23 enzymes. Furthermore, the analysis hints that LytU and LytM is ancestral for the defined group of bacteriocins, but taking into account the relatively small size of this group (Figure 2, panel A), observations regarding ancestry should be treated with caution.

Finally, there are M23 family members that do not display a complete set of catalytic residues and/or lack zinc, but retain the M23 fold. In most cases, these proteins are involved in protein–protein interactions, which is the case of EnvC and NlpD cell division proteins of *E. coli* (Uehara et al., 2009; Peters et al., 2013). It is worth stressing that the sole mammalian M23 member described so far, LECT2, displays a degenerative fold that lacks catalytic histidine and contains an additional loop protruding from the active centre (Zheng et al., 2016). LECT2 is expressed predominantly in the liver and acts as a tumor suppressor in hepatocellular carcinoma. To summarize, degenerative M23 folds present a good example of the gradual impairment in the sequence leading to the evolution of new biological functions.

### Active site and groove architecture

**Figure 3:**
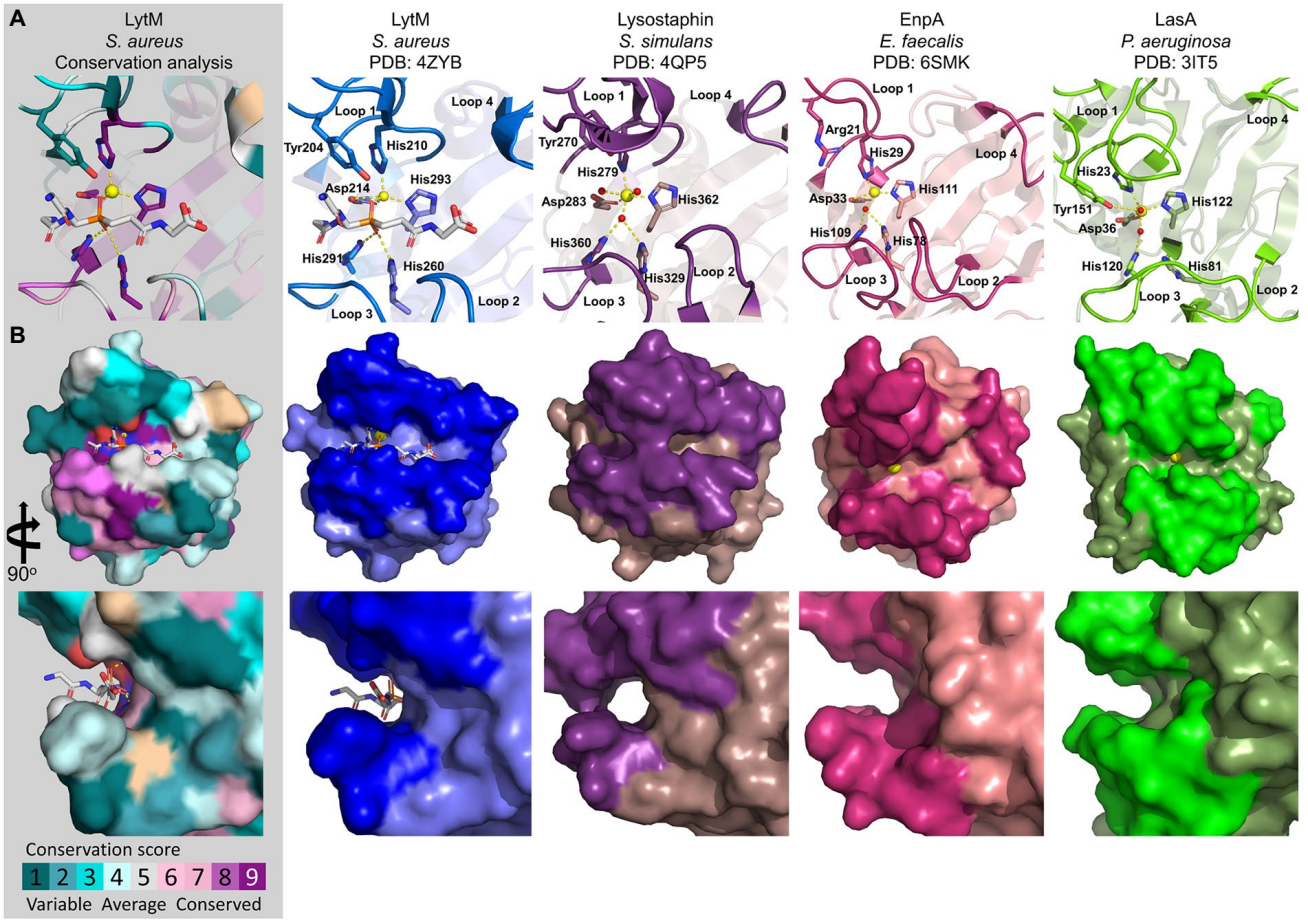
Crystal structures of representative M23 metallopeptidases: LytM (blue; 4ZYB), Lysostaphin (violet; 4QPB), EnpA (magenta; 6SMK), and LasA (green, 3IT5). The M23 peptidases sequence conservation score was calculated in the ConSurf server and is displayed on LytM surface (PDB ID: 4ZYB; Landau et al., 2005). **(A)** Active sites and loop architectures are represented as cartoons. The active site residues are depicted as bold sticks and labelled accordingly. The zinc ion is shown as a yellow sphere. The LytM was crystallized in the presence of tetraglycine phosphinate, and the resulting complex served as a model for the substrate-binding mechanism. **(B)** The top view (upper panel) and site-zoomed view (bottom panel) of the corresponding surface represent the M23 structures.

M23 peptidases have a prominent amount of structural data available that provides insights into their catalytic mechanisms (Table 1, Figure 3). Here we focus on four PGHs from the M23 family, namely LytM, Lss, EnpA, and LasA, whose structures provide detailed information on the overall fold, active site, and loops architectures of M23 peptidases (Firczuk et al., 2005; Spencer et al., 2010; Sabala et al., 2014; Grabowska et al., 2015; Małecki et al., 2021).

PGHs of the M23 family comprise a β-sheet core that serves as a rigid bottom of a substrate-binding groove. Residues of the conserved motifs form an active site with zinc ion in the centre and are coordinated by two histidine and one aspartic acid residues that comprise the conserved motifs: **H**(x)_n_**D** and Hx**H**. The first histidine residue of the HxH motif is not a Zn(II) ligand and is proposed to coordinate and activate incoming water molecule, which triggers the nucleophilic attack on the scissile bond. Another histidine residue that is located in a consensus sequence of ca. 30 amino acids before the HxH motif (e.g., His260 in LytM, His329 in Lss, His78 in catalytic domain of EnpA and His81 in LasA) is also located in the vicinity of the active water molecule; therefore, it might participate in its activation. Other residues important for the catalysis are Tyr204 in LytM, Tyr270 in Lss, and Tyr151 in LasA, which stabilize the oxyanion intermediate of the cleavage reaction. For EnpA_,_ the same role was proposed for Arg21, which confers additional stabilization of other residues, therefore, sustaining proper active site geometry. Arginine at the corresponding position is also present in Csd3, whereas in gp13, arginine is replaced by a glutamine residue (Xiang et al., 2008; An et al., 2015).

In contrast to a very conserved active site, the architecture of the entire active groove is determined by variable loops (L1–L4; Figure 3A). The arrangement of loops influences the shape, and therefore, determines the specificity of the particular enzyme (Małecki et al., 2021). Indeed, PGHs from the M23 family preferentially digest pentaglycine (LytM, LytU, and Lss) and display elongated, deep, and narrow grooves (Sabala et al., 2014; Grabowska et al., 2015). In contrast, EnpA and LasA, display a more open site at the substrate entrance, which enables them to accommodate a wider range of substrates, for instance, serine (EnpA) or large, aromatic residues (LasA; Figure 3B; 31, 36).

### Isoelectric point

Most bacterial surfaces are negatively charged, whereas antimicrobials in general are basic molecules/peptides/enzymes. It is reasoned that their net charge serves as a means to alleviate the effect of electrostatic repulsion and enable them to approach the bacterial cell envelope (Low et al., 2011). Cationic antimicrobial peptides (CAMPs) or daptomycin are examples of antimicrobial agents, whose basic charge helps them access their cellular target, namely the cell membrane (Jones et al., 2008; Weidenmaier and Peschel, 2008).

This phenomenon has been exploited on PGHs in several enzyme engineering studies. By altering the net charge of the enzymes, their lytic activity can be improved (Low et al., 2011; Díez-Martínez et al., 2015). Furthermore, rather than global, net charge alteration contributes to the enhancement of the lytic action of PGHs (Shang and Nelson, 2019; Zhao et al., 2020).

Two novel M23 peptidases with different isoelectric points (pI) were identified in *S. pettenkoferi* (Wysocka et al., 2021, 2022). One of the enzymes was acidic (theoretical pI = 5.68) and the other one was basic (theoretical pI = 10.28). In literature, basic M23 peptidases have been described, for instance, Lss or LytM with pI = 9.10 and 7.99, respectively, along with acidic M23 peptidases, such as gp13 (theoretical pI = 5.31) from bacteriophage phi-29. Apart from these few examples, knowledge concerning the net charge of M23 peptidases and their role in their activity is limited. Therefore, we filled this gap by calculating the theoretical pI of a large set of M23B peptidase deposits present in the InterPro database. The search was narrowed down to bacterial M23 peptidases that contain highly conserved zinc-binding sequence motifs. InterPro defines the M23 domain from the Hx_3_D up to the HxH motif, therefore, these annotations do not include the loops 1 and 4 regions that form the enzyme’s active groove (Sabala et al., 2014). Therefore, we extended the region defined for the analysis with 25 amino acids (aa) at each overlapping side of the domain. Ultimately, the average size of the M23 peptidase in the analyzed set of 90,000 protein sequences was 131 ± 3.3 aa.

**Figure 4:**
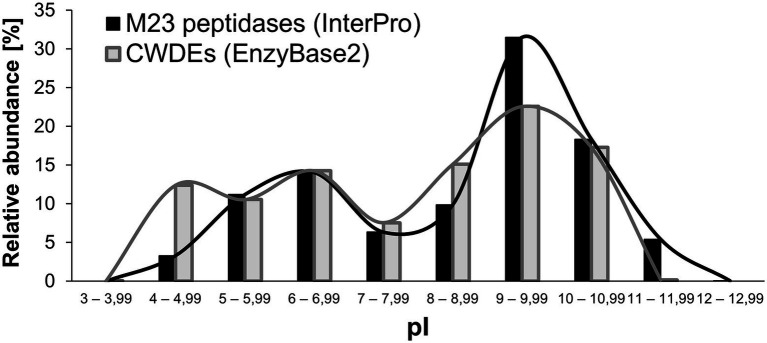
Calculated isoelectric points of M23B bacterial peptidoglycan hydrolases in comparison to the isoelectric points of cell-wall degrading enzymes (CWDEs) gathered in the EnzyBase2 database. Values are presented both in chart bars and lines, the latter serves to illustrate the presence of maxima in each dataset.

The results of the calculation demonstrated that the distribution did not follow the normal Gaussian curve (Figure 4). In our interpretation, this was the first indication that the net charge of the enzymes plays an important regulatory function in their performance. The distribution had two maxima: a smaller one at a pI of 6–6.99 and a higher one at a pI of 9–9.99. We observed that the majority of the M23 domains had a basic pI (~65% of all analyzed domains). Interestingly, 35 domains had a theoretical pI that was higher than 12. M23 domains with an acidic pI comprised ~29% of the probed dataset. None of the domains had a pI less than 4. Only ~10% of the deposits were found around the neutral pI (7–7.99). In our interpretation, this small fraction reflected the fact that the net charge of the M23 peptidases was altered to omit the physiological pH. A similar analysis was done previously for a broad set of cell wall-degrading enzymes (CWDEs) collected in the EnzyBase2 database, which exhibited a similar pattern with maxima at acidic and basic pI ranges, where the latter fraction was a dominant one (Wu et al., 2012). These two maxima have been found for both M23A and M23B subfamilies, and no major differences in pI distribution is observed for autolysins and bacteriocins (data not shown). Therefore, the net charge distribution in the M23 peptidases that we found was consistent with what is defined for PGHs in general. However, it should be noted that we calculated only the pI of the M23 domains and not the protein as a whole as in the EnzyBase2 database (Wu et al., 2012, 2). Identification of maxima of the distribution of net charges in PGHs was in clear contrast to what is already known about prokaryotic proteomes. For instance, analysis of ca. >4,000 proteins in *E. coli* revealed a distinct Gaussian distribution of pI values (Sear, 2003). It is tempting to speculate that the observed two-peak distribution of net charges was characteristic of the enzymes involved in PG digestion, which necessitates further research.

**Figure 5:**
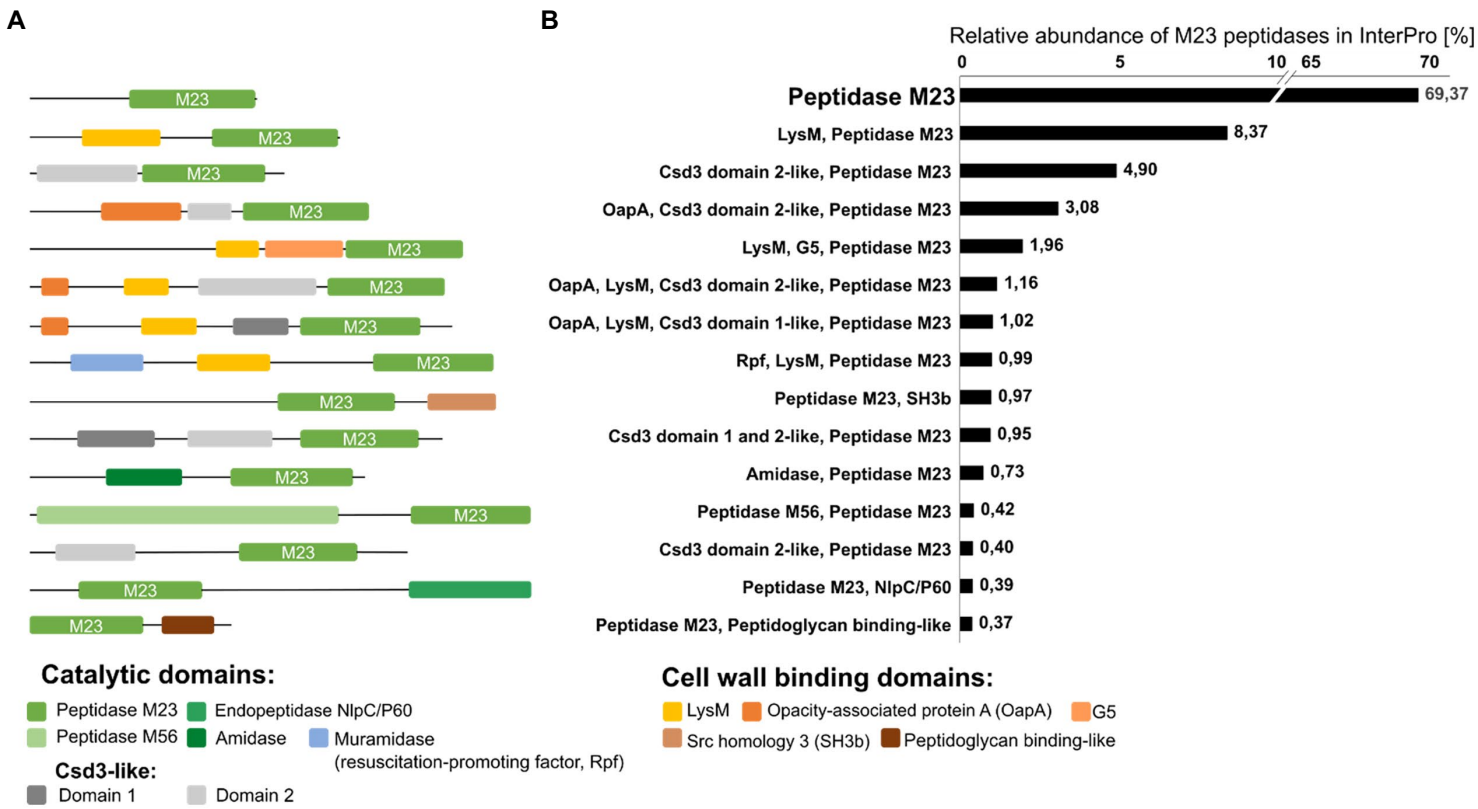
Schematic representation of the most common architectures of bacterial peptidoglycan hydrolases containing the M23 domain (A) and their relative abundance in percentage [%] among the peptidase M23 family of proteins in the InterPro database (over 90,000 sequences with at least 37% amino acid sequence identity of M23 peptidase domain with the lysostaphin M23 domain were included in the analysis).

### Domain architecture of PGHs with the M23 domain

A common feature of PGHs is their modular architecture that combines different catalytic domains (CATs; known also as enzymatically active domains, EADs) and cell wall-binding domains (CBDs; or cell wall-targeting domains, CWTs; Figure 5). In Gram-positive bacteria, the most common CATs are CHAP (cysteine, histidine-dependent amidohydrolases/peptidase), NlpC-P60 (new lipoprotein C/protein of 60 kDa), glucosaminidases and M23 peptidases, whereas, in Gram-negative bacteria – the Lyz-like (lysozyme-like) and M23 peptidase domains are important (Vermassen et al., 2019; Mitchell et al., 2021).

The development of new domain combinations is an important mechanism in protein evolution (Marsh and Teichmann, 2010). Often, they contain two catalytic units with different specificities towards PG bonds (peptidase, amidase, or glycosidase) and the cell wall-binding domain (Vermassen et al., 2019). Available literature revealed that M23 catalytic domains are commonly found in such multi-domain enzymes. For instance, mature lysostaphin comprises an N-terminal M23 domain linked by a short flexible peptide to SH3b (Src Homology 3 bacterial type)-binding domain. Zoocin A from *Streptococcus equi* subsp. *zooepidemicus* 4,881 is another example that contains the TRD domain (target recognition domain) located C-terminally to the M23 catalytic domain (Lai et al., 2002; Sabala et al., 2014).

To gain insights into a diversity of M23 peptide architectures, we performed database analysis on the same set of M23 peptidase deposits as done previously for the pI analysis. InterPro database search for M23 peptidases resulted in a list of over 1,000 different domain architectures of enzymes containing the M23 peptidase domain Figure 5.

Almost 70% of the deposited M23 sequences were annotated as a single-domain protein with long N-terminal sequences that could be involved in protein secretion and/or activity regulation. The second most abundant group was M23 peptidases with LysM (lysin motif), a cell wall-binding domain that recognizes GlcNAc in peptidoglycan. Multiple LysM sequences in the N- or C-terminus or between domains are often observed in PGHs, however, only one or two LysM positioned at the N-terminus are typical for M23 peptidase-containing hydrolases. They represent almost 10% of the total M23 domain architectures. M23 peptidases are often found with Csd-3-like domains, particularly with domain 2. Csd3 protein (also known as HdpA) is a LytM-like M23 peptidase from *Helicobacter pylori*, which is involved in cell shape determination. In addition to the C-terminal catalytic domain, Csd3 contains two domains, domains 1 and 2. Domain 1 occludes the active site of the M23 catalytic domain, thus playing an inhibitory role, whereas domain 2 is held against the M23 domain by the C-terminal tail region that protrudes from the M23; however, the exact role of domain 2 is not known (An et al., 2015). OapA (opacity-associated protein A) domain or its N-terminal fragments have also been found with M23 and Csd-3-like domain 2. OapA was first described in *Haemophilus influenza* as a factor that confers colony opacity and pathogen attachment to human conjunctival epithelial cells (Prasadarao et al., 1999). The OapA domain of the *E. coli* protein, YtfB, recognizes PG (Burke et al., 2013), therefore, it can be considered as cell wall-binding domain. Some enzymes containing the M23 peptidase harbor the G5 domain located between the LysM and the M23 domain. G5 domains are widely distributed in bacteria, especially among streptococcal strains and are involved in biofilm formation. It was suggested that they could recognize N-acetylglucosamine (Bateman et al., 2005), but further studies have contradicted that assumption (Paukovich et al., 2019).

Another cell wall-binding domain that co-occurs with the M23 peptidases is the SH3b domain. This domain has been defined for lysostaphin and early reports revealed that it confers its selectivity towards *S. aureus* pentaglycine cross-bridges (Tossavainen et al., 2018). Further biochemical and structural research on SH3b domains revealed their potential to bind PG stem-peptides and other CW components, including teichoic acids and serum components (Mitkowski et al., 2019; Gonzalez-Delgado et al., 2020; Shen et al., 2020).

Some enzymes that contain the M23 peptidase domain also contain other catalytic domains, but they comprised less than 1% of all hits in the database. Among these were enzymes with the catalytic Rpf-like domain, amidase domain, M56 peptidase, and NlpC/P60. Rpfs represent bacterial cell wall lytic enzymes called resuscitation-promoting factors that enable bacteria to exit their dormant state and return to active growth. These enzymes share structural homology with lysozyme and lytic transglycosylases and present muralytic activity (Cohen-Gonsaud et al., 2004, 2005; Sexton et al., 2020). Amidases can separate the glycan chain of PG from the stem peptide (Herbold and Glaser, 1975). NlpC/P60 proteins are a well-known class of cell wall hydrolases that typically cleave the linkage between D-Glu and Dpm (or Lys) within the PG stem peptides (Anantharaman and Aravind, 2003; Kim et al., 2020), whereas the role of M56 peptidase in PG cleavage has not been shown. The M56 clan is represented by BlaR1 and MecR1 which function as parts of the signal transduction systems that trigger bacterial resistance to β-lactam antibiotics (López-Pelegrín et al., 2013).

Taken together, enzymes comprising M23 peptidase domains display prominent architectural variety. Although the most common architecture represents a single catalytic domain, the additional catalytic or binding domains often contribute to higher activity and even broader specificity (Mao et al., 2013).

### Latency mechanisms

Due to the potentially detrimental effect that PG hydrolases pose on the cell wall integrity, their action is maintained under tight control. One of the means to limit their action takes place at the expression level and is often found in PGHs that are involved in cell division (Uehara and Bernhardt, 2011). A common architectural theme of M23 peptidases is the presence of pro-peptides, which have been found in LasA (*P. aeruginosa*), ALE-1 (*S. capitis*), Lss (*S. simulans*), LytM (*S. aureus*), and SpM23 enzymes (*S. pettenkoferi*). It is usually the N-terminally located region that occupies the substrate space in the active groove. Most often, pro-peptides display tandem architecture, comprising repeated motifs and low similarity to currently known domains. Some pro-peptides undergo proteolytical digestion (Lss, LasA), but this is not always the case (ALE-1, Spm23_A; Sugai et al., 1997; Thumm and Götz, 1997; Kessler et al., 1998; Wysocka et al., 2021). In addition, LytM was found to be proteolytically activated *in vitro* (Odintsov et al., 2004), but in the proteomic analysis of *S. aureus* cellular extracts, LytM was identified solely in its full-length, latent form (Pieper et al., 2006).

Several M23 peptidase structures described so far detail the inhibitory mechanisms posed by pro-peptides. In the latent form of LytM, the pro-region has a physically protruding active site, and its arrangement allows the carbonyl oxygen of the Asn117 side chain to reach catalytic Zn(II), thereby substituting for catalytic water. This mechanism has been termed the “asparagine switch” in connection to the cysteine switch defined for pro-matrix metalloproteases (Van Wart and Birkedal-Hansen, 1990).

**Figure 6:**
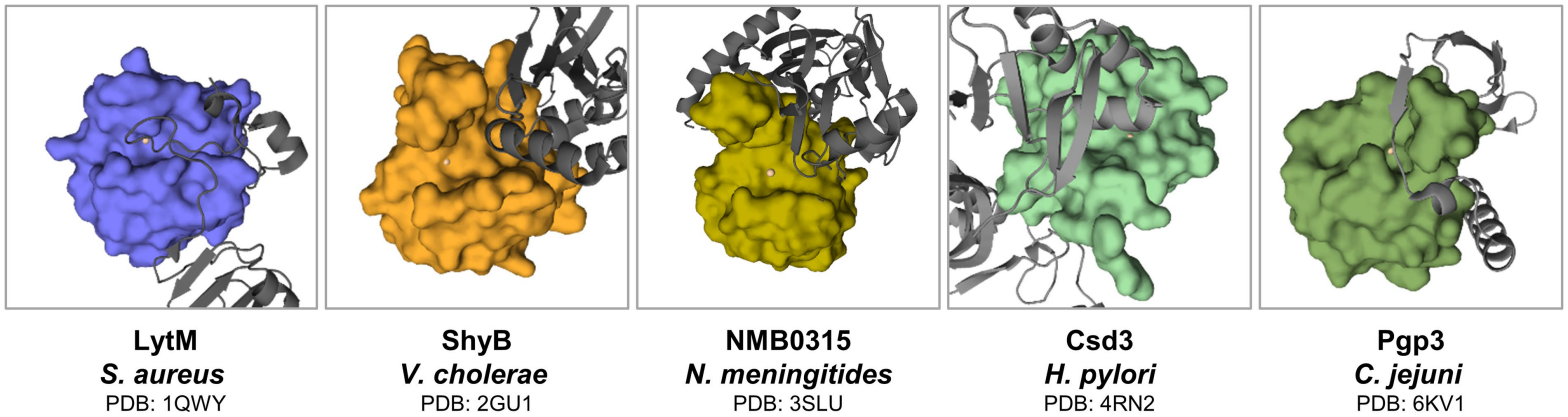
Latency forms of M23 peptidases. Regions that protrude from the active site are indicated in gray. M23 domains were defined accordingly in literature (Odintsov et al., 2004; Ragumani et al., 2008; Wang et al., 2011; Min et al., 2020) as depicted in surface representation. Active site metals are presented as spheres.

In the case of the LytM latent form, inhibition is provided by the long flexible loop (Figure 6). In contrast, mechanisms of latency defined for other M23 family members differ at the level of secondary structures involved. The structure of full-length ShyB (Vly) in *Vibrio cholerae* (Ragumani et al., 2008) demonstrated that the active groove of peptidase is occupied by the N-terminal helix. Catalytic centre residues of Domain III of NMB0315 (*Neisseria meningitides*) are implicated in multiple contacts with the short loop of domain I (Wang et al., 2011). Pro-region of Csd3 (also known as HdpA) comprises two domains, and the occluding helix of Domain 1 enters the active site (An et al., 2015). *Helicobacter pylori* is associated with various gastrointestinal diseases such as gastritis, ulcers and gastric cancer. Its colonization of the human gastric mucosa requires high motility, which depends on its helical cell shape. Seven cell shape-determining genes (*csd1*, *csd2*, *csd3/hdpA*, *ccmA*, *csd4*, *csd5* and *csd6*) have been identified in *H. pylori* (An et al., 2016). Their proteins play key roles in determining the cell shape through modifications of the cell-wall peptidoglycan by the alteration of cross-linking or by the trimming of peptidoglycan muropeptides. Among them, Csd3 is a bifunctional enzyme. Its D,D-endopeptidase activity cleaves the D-Ala(4)-mDAP(3) peptide bond between cross-linked muramyl tetrapeptides and pentapeptides. It is also a D,D-carboxypeptidase that cleaves off the terminal D-Ala(5) from the muramyl pentapeptide. The crystal structure of this protein has been determined, revealing the organization of its three domains in a latent and inactive state. The N-terminal domain 1 and the core of domain 2 share the same fold despite a very low level of sequence identity, and their surface-charge distributions are different. The C-terminal LytM domain contains the catalytic site with a zinc ion, like the similar domains of other M23 metallopeptidases. Domain 1 occludes the active site of the LytM domain. The core of domain 2 is held against the LytM domain by the C-terminal tail region that protrudes from the LytM domain (Odintsov et al., 2004). Lastly, Pgp3 of *Campylobacter jejuni* can adopt two conformations: open and closed. In the latter one, loop L1 from the flexible linker that joins its catalytic and binding modules enters the groove and, therefore, regulates the catalysis of Pgp3 (Min et al., 2020). To summarize, the presence of pro-region is a common feature of the M23 peptidases. Although the great variability in its sequence and mechanisms of active site blocking indicates that latency mechanisms are not exactly conserved across this enzymatic family.

## Physiological functions of M23 peptidases

The major fraction of the M23 deposits in our dataset came from bacterial species (97% according to the InterPro database), which indicates that they are predominantly bacterial proteins (Blum et al., 2021). The remaining M23 peptidase domains deposited in this database were distributed among viruses, archaea, and eukaryotes. To date, no M23 peptidase has been found in fungi.

**Figure 7:**
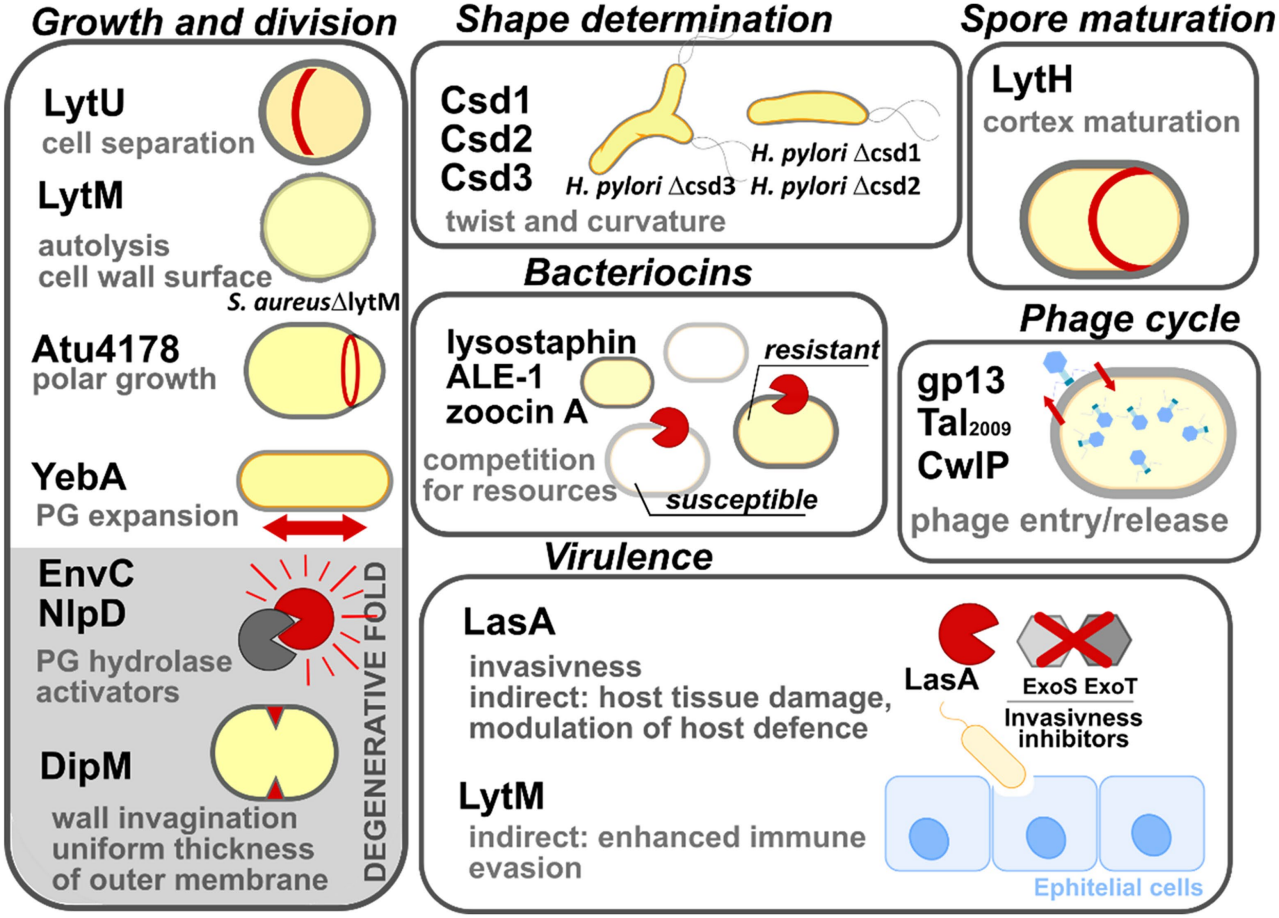
Multiple biological roles of M23 peptidases. The enzymes are grouped here according to their biological roles: growth and division, shape determination, spore maturation, intraspecies competition (bacteriocins), phage lytic cycle, and bacterial virulence. M23 family members that display degenerative folds are shown on the gray background. All abbreviations used have been explained in the text.

There are many examples demonstrating the diversity of biological functions of M23 peptidases; they are involved in bacterial physiology, including involvement in the processes of cell growth, division, or competition for resources (Figure 7). Furthermore, some M23 peptidases display degenerative folds that remain functional. Detailed examples are listed below.

### Growth and division

The cell cytoplasm is surrounded by polymerized PG sacculus, whose digestion is a prerequisite for cell expansion, and ultimately, cell growth and division. The means for this digestion are enzymes that digest PG and are often termed autolysins or simply PGHs (Uehara and Bernhardt, 2011). Autolysins are a highly redundant group. Therefore, in most cases, not single enzymes, but several autolysins are required for bacterial growth and division collectively (Heidrich et al., 2002; Bisicchia et al., 2007; Singh et al., 2012; Wheeler et al., 2015). Due to the potentially detrimental action of autolysins on cell integrity, their lytic activity is tightly controlled temporally and spatially. They have thus been termed pacemakers of bacterial growth (Höltje, 1995).

Several M23 peptidases act as PGHs in this process. LytU of *S. aureus* participates in mother-daughter cell separation, which was inferred from the presence of a “scar” that marks the previous division plane in a mutant lacking the corresponding gene (Raulinaitis et al., 2017). Another *S. aureus* M23 peptidase, LytM, was identified in an autolysis defective mutant (Mani et al., 1993), which displayed increased roughness of the cell envelope (Mani et al., 1994). Atu4178 of the plant pathogen *Agrobacterium tumefaciens* confers cell elongation and is essential for the polar growth of the cell (Figueroa-Cuilan et al., 2021). YebA, together with Spr and YdhO (NlpC/P60 peptidase superfamily), are redundantly essential for the growth of *E. coli*. Their absence causes the incorporation of the nascent PG fragments to stall, triggering bacterial cell lysis (Singh et al., 2012).

Lastly, M23 peptidases that lack catalytic motifs and/or zinc are termed degenerate folds. Interestingly, although they exhibit function that is different from that of the M23 family enzymes, they remain functional and are involved in the growth processes. Several studies have shown that degenerate folds act as the protein hubs that are involved in many interactions with division machinery components and are important at the later stages of cell division (Zielińska et al., 2017; Figueroa-Cuilan et al., 2021). For instance, EnvC and NlpD activate amidases, which are lytic agents involved in cell separation (Uehara et al., 2010). Direct interaction between the catalytically inactive groove of EnvC or NlpD and the amidase, disrupts the autoinhibitory state of the latter (Uehara et al., 2009; Peters et al., 2013). DipM of *Caulobacter crescentus,* which is an orthologue of EnvC and NlpD, was found to be bound at the division site; it contributed to the invagination of the outer envelope; it also confers a uniform PG thickness, and consequently, ensures the correct morphology of the *C. crescentus* outer membrane (Goley et al., 2010; Möll et al., 2010).

### Shape determination

Csd3 (HdpA) is the M23 peptidase that is responsible for the curved and twisted shape of *H. pylori* (Bonis et al., 2010). Deletion of its gene results in cell branching, whereas its overproduction leads to cell rounding. *H. pylori* Δ*csd3* mutant has been ineffective in stomach colonization, which demonstrates that its cell shape is an important factor contributing to the survival of *H. pylori* in the viscous environment of the gastric mucosa. Further research revealed that the shape of this pathogen depends on the action of other M23 peptidases, namely Csd1 (PGH) and Csd2 (degenerate protein), which act as heterodimers (Sycuro et al., 2010; An et al., 2016). Once their genes are deleted, cells are less curved and twisted, which impairs stomach colonization. Since Csd1 and Csd2 are conserved across Gram-negative bacteria, they may act in similar manners in other species (Sycuro et al., 2010).

### Spore maturation

Spores are dehydrated structures synthesized by certain members of phylum Firmicutes; they are responsible for conferring persistence and survival abilities to bacteria under unfavorable conditions (Ellar, 1978). They are separated from the cells by an altered cell wall termed the cortex. A unique feature of the cortex is a low cross-linking level of its PG, which is mediated by LytH in *Bacillus subtilis*. Its gene expression is limited to the sporulation phase alone. LytH digests the bond within stem peptide, leading to enrichment of MurNAc substituted with single L-Ala, which is not a suitable substrate for a cross-linking reaction (Horsburgh et al., 2003).

### Competition for resources (Bacteriocins)

Several M23 peptidases act as the warhead to eliminate competitors of their bacterial host, which reside in the same ecological niche. The best-studied enzyme with this type of function is lysostaphin (Heng et al., 2007). A characteristic feature of this group of enzymes is that they leave their producer intact. In the case of lysostaphin, this is achieved by a few means, including modification of *S. simulans* PG, making it lysostaphin-resistant (Thumm and Götz, 1997); the high specificity of this enzyme is conferred by its binding domain (Baba and Schneewind, 1996; Gründling and Schneewind, 2006; Mitkowski et al., 2019). Further studies revealed more examples of lysostaphin types, namely ALE-1 (*Staphylococcus capitis* (Sugai et al., 1997)) and zoocin A (*Streptococcus equi* subsp. *zooepidemicus* 1884 (Gargis et al., 2009)) that likely act as bacteriocins as well.

### Phage cycle

M23 peptidases are leveraged by bacterial viruses (hereafter: phage) to enter bacterial cells (virion-associated lysins). At the late stages of their cycle, they also help release their progeny (endolysins). For instance, gp13 of *B. subtilis* phage varphi29 and Tal_2009_ found in *Lactococcus lactis* prophage region are both located at the tip of the phage tail-knob and facilitate the insertion of the genetic material of the phage into the bacterial prey by digesting the cell wall PG (Kenny et al., 2004; Cohen et al., 2009). The CwlP identified in *B. subtilis* prophage SP-*β* also acts as a virion associated lysin which was inferred from its genetic environment (proximity to phage tail as a measure of the protein domain; Sudiarta et al., 2010). The endolytic function was assigned to SpAE, a multidomain enzyme-containing M23 peptidase domain, identified in staphylococcal phage 2638A. However, deletion of the M23 domain revealed that major lytic activity of SpAE lies within its amidase, not the peptidase domain (Abaev et al., 2013; Proença et al., 2015). Many M23 domain-containing proteins have been assigned as endolysins or putative endolysins in PhaLP database (Criel et al., 2021) but the real endolytic function of M23 peptidase has to be confirmed *in vitro*.

### Virulence

Some M23 peptidases are leveraged as virulence factors by certain pathogens. For instance, LasA of *P. aeruginosa* degrades protein factors (ExoS, ExoT) that inhibit bacterial entry to the epithelial cells (Cowell et al., 2003), and also stimulates the elastolytic activity of LasB, which triggers host tissue damage (Fleiszig et al., 1997; Kessler et al., 1997). Additionally, LasA modulates the host defense system by impacting syndecan-1 shedding of various host cell types (Park et al., 2000, 2001). Moreover, the deletion of *lytM* in *S. aureus* cause impairment of its virulence which was observed in the rat endocarditis model (Mani et al., 1994). Further research studies revealed that attenuated virulence of *S. aureus* LytM^−^ is consistent with the decreased levels of staphylococcal protein A (SpA; Becker et al., 2014), which affects the immune evasion potential of this pathogen (O’Halloran et al., 2015). It must be noted that LytM, on its own, potentiates a strong antibody response, therefore, it can be considered to be used as the antigen in the vaccines that protect against refractory *S. aureus* infections (Wang et al., 2021).

Degenerative M23 folds may also contribute to pathogenicity. For example, cells of *Yersinia pestis* that are devoid of NlpD were chained, as well as displayed heat sensitivity and an inability to disseminate into the mice organs (Tidhar et al., 2009, 2019). Due to the highly attenuated phenotype, *Y. pestis* Δ*nlpD* was further explored as a promising vaccine candidate against plague (Wu et al., 2018).

## M23 peptidases as antibacterial weapons

The discovery and introduction of penicillin in the 1940s revolutionized the medical field and saved millions of human lives. Penicillin initiated the golden age of antibiotics that lasted until the beginning of the 1980s (Hutchings et al., 2019). However, it did not take long to identify the first antibiotic-resistant strains that did not respond to its treatment (Antimicrobial Resistance Collaborators, 2022). The misuse and overuse of antibiotics in healthcare, animal breeding, and plant agriculture, have led to the rapid spread of antimicrobial resistance (AMR) and the emergence of multi- and pan-resistant variants termed ‘superbugs’. World Health Organization (WHO) declared AMR as one of the top 10 global public health threats. Therefore, there is an urgent need to develop novel compounds that are effective against antibiotic-resistant organisms. PGHs are regarded as very promising in that respect. PGHs are deemed to be a novel class of antimicrobials: enzybiotics (enzyme-based antibiotics; Nelson et al., 2001). Their main advantages are high specificity, selectivity, rapid mode of action, and low probability of the development of resistance. They are effective against biofilms which are of great value in the treatment of chronic and refractory infections that are caused by multidrug-resistant pathogens (Dams and Briers, 2019).

### Lysostaphin – A weapon against *Staphylococcus aureus*

Lss was discovered in the 1960s and gained prominent attention from the scientific community early on. It remains the best-studied enzybiotic of the M23 family. Early reports proposed its use for rapid detection tests (Severance et al., 1980), but it was quickly introduced to clinics to treat patients suffering from resistance to antibiotic treatment of *S. aureus* infection (Stark et al., 1974; Kokai-Kun, 2012). Several companies exchanged their licenses for patents and optimized the large-scale production of this enzyme (Kokai-Kun, 2012). Although Lss proved very effective to eradicate *S. aureus in vivo* (Schaffner et al., 1967), the research on Lss has stalled for years. Major concerns were related to its immunogenicity (Kokai-Kun, 2012), which became pronounced upon repeated dosing, leading to an increase in Lss-neutralizing antibodies (Schaffner et al., 1967; Harrison et al., 1975). Currently, several approaches are available to successfully address this issue, such as limiting treatment to a single dose, combining Lss with polyethylene glycol, or deimmunization of Lss *via* site-directed mutagenesis of its epitopes (Kusuma et al., 2007; Zhao et al., 2020). Recently, an Lss variant, F12, was designed, which carries 14 aa-long substitutions, leading to diminished immunogenic response without a loss of its lytic activity against methicillin-resistant *S. aureus* (MRSA; Zhao et al., 2020). Another strategy to conquer the immunogenicity of Lss is its immobilization, which limits its uncontrolled diffusion. This is particularly useful to treat superficial infections of *S. aureus,* leading to chronic wound development (James et al., 2008). Upon covalent attachment of Lss to chitosan-cellulose nanofibers, the enzyme remained effective, as demonstrated by the complete eradication of the *S. aureus* in the skin infection model (Miao et al., 2011).

Although most PGHs display a low propensity for resistance development, rapid resistance against Lss was observed under laboratory conditions; a single high-dose exposure was enough to identify insensitive clones (Zygmunt et al., 1967). The activity of Lss is strongly affected by the insertion of a serine residue in the staphylococcal cross-bridge, which is commonly found in staphylococci (Zygmunt et al., 1968; Grishin et al., 2020). Resistance to Lss was found in MRSA, where it relied on a different mechanism, whereby a single-glycine cross-bridge was formed. However, this mutation reduces fitness, virulence, and re-sensitizes this pathogen to β-lactams. So far, this phenotype has been identified only under laboratory conditions, and no variants of this type have been isolated from the environment or clinical settings (Grishin et al., 2020). Many studies have concentrated on the use of Lss in combination with other antimicrobials, including antibiotics, enzybiotics, and antimicrobial peptidases (Polak et al., 1993; Becker et al., 2008; Desbois and Coote, 2011; Schmelcher et al., 2012). Treatment of MRSA with Lss and β-lactams causes synergistic or additive effects, depending on the antibiotic type, which has proven to be effective both *in vitro* and *in vivo* (Polak et al., 1993; Hertlein et al., 2014). Additionally, this approach decreases the probability of the development of resistance to Lss (Climo et al., 2001).

Synergistic effects of Lss against MRSA were observed by combining it with other enzybiotics, which is likely due to the change in PG structure by one of the lytic enzymes, that favors the action of another (Becker et al., 2008). Lss was combined with engineered phage lysins that target the D-Glu-L-Lys bond in the stem peptide, which successfully treated mastitis in a mouse model (Schmelcher et al., 2012). Lss also synergizes with cationic antimicrobial peptides (CAMPs; Desbois and Coote, 2011), which is likely due to the ability of the latter to easily penetrate disrupted PG to reach its cellular target (membrane; Wittekind and Schuch, 2016). Finally, Lss is effective against *S. aureus* that invades eukaryotic cells, indicating that it can act optimally under conditions that are typical of intracellular compartments (cytoplasm, phagolysosomes). It can thus be used to effectively treat extracellular pathogens by either its fusion with protein transduction domains (PTDs) or other lytic domains (Becker et al., 2008, 2016). Overall, although several concerns regarding the use of Lss have been raised, many issues regarding its safety have been already solved. Therefore, owing to its many unique and attractive features, even after 60 years of its discovery, Lss remains a promising antimicrobial agent to treat refractory MRSA infections.

### Chimeric enzymes

A common practice regarding the development of new enzybiotics is to employ enzyme engineering to optimize their action. The modular architecture of PGHs creates a perfect opportunity to generate novel and improved variants that display the desired lytic activity or enhanced stability. This has been exemplified successfully in several studies that employed domain switching strategy or linker improvement (Proença et al., 2015; Becker et al., 2016; Eichenseher et al., 2022).

This strategy enhanced the lytic activity of certain enzymes containing the M23 peptidase domain. Upon truncation of the N-terminal pro-region of full-length LytM, its catalytic domain displayed lytic action against *S. aureus,* leading to its commercialization (Auresine®) and use for laboratory purposes.

Auresine® was engineered by fusing the catalytic domain of the Lss with the SH3b cell wall-binding domain (M23_LytM_-SH3b_Lss_). The resulting chimeric enzyme (AuresinePlus) displayed improved lytic activity under physiological conditions, such as high ionic strength and pH (Osipovitch and Griswold, 2015; Jagielska et al., 2016). The same approach was implemented on LytU, which upon fusion to SH3b_Lss_, was effective against *S. aureus* at lower concentrations as reflected in a significant decrease in its MIC value as compared to the MIC of LytU alone (421 times lower; Taheri-Anganeh et al., 2019).

Another chimeric enzyme is Staphefekt SA.100. It contains an M23 domain derived from lysostaphin, MurNAc-L-Ala amidase (Ami), linked to the SH3b cell wall-binding domain from staphylococcal phage endolysin, Ply2638. Furthermore, the linker has been improved (deletion of 44 aa between the M23 and the Ami domains) and the resulting version, which is called XZ.700, displays enhanced antibacterial action against *S. aureus* (MIC value ∼75 nM for XZ.700, whereas 350 nM for SA.100; Eichenseher et al., 2022). Staphefekt™ from Micreos, is the active compound of Gladskin products and is applied in the form of a cream that alleviates the severity of atopic dermatitis symptoms. Case studies have revealed that Staphefekt™ is effective in controlling, rather than eradicating methicillin-resistant *S*. *aureus* (MRSA) that causes dermatoses. The company has already announced the launch of phase III for the treatment of eczema.^1^

Extensive studies have been carried out on PGHs and their engineered versions, which have led four candidates to launch seven clinical trials, all of which concern the development of treatments against *S. aureus* infections. Three candidates presented positive outcomes, namely P128 (StaphTAME), Lysin *CF*-301 (Exebacase), and Staphefekt™ SA.100; the latter is a chimeric enzyme-containing M23 peptidase domain.^2^ Progress of pre-clinical studies on enzybiotics and their results are updated in several databases, such as phiBiOTICS (Hojckova et al., 2013), EnzyBase (Wu et al., 2012), or BACTIBASE (Hammami et al., 2010).

Computational tools are also useful in terms of the development of new enzybiotics. They are being developed to identify and classify new peptidoglycan hydrolases based on genomic and metagenomic data (HyPe—A Peptidoglycan Hydrolase Prediction Tool; Sharma et al., 2016). The introduction of the VersaTile technique, a DNA assembly method for the rapid building of combinatorial libraries of engineered lysins, led to the construction of approximately 10,000 lysin variants comprising four main modules: catalytic domain, cell wall-binding domains, linker and outer membrane permeabilizing peptides (OMPs, peptides commercialized as Artilysin®; Gerstmans et al., 2016). High-throughput screening procedures led to the identification of a new variant with high antibacterial activity against *Acinetobacter baumannii* in human serum (Gerstmans et al., 2020). Unfortunately, Artilysin-based enzymes often do not show activity under physiological conditions, e.g., in the human serum (Thandar et al., 2016; Larpin et al., 2018). Recently, another type of chimeric enzyme was developed called lysocins by combining lysins with bacteriocins, which can provide periplasmic import. Lysocins are composed of bacteriocin, pyocin S2 (PyS2), responsible for surface receptor binding and outer membrane translocation. The GN4 lysin can hydrolase β-1,4 glycosidic bond between MurNAc and GlcNAc displaying antipseudomonal activity in human serum, which efficiently disrupts biofilms (Heselpoth et al., 2019).

## Conclusion

M23 peptidases are common prokaryotic PGHs. Over the years many studies have exemplified their specificity, mode of action, structural features, and physiological functions. Now, this knowledge can serve practical purposes, particularly for the development of new antimicrobial agents. This beneficial feature can be explored in two different ways. Firstly, due to their potent bactericidal action, PGHs can directly kill bacteria, including drug-resistant variants, and contribute to the eradication of refractory infections. Moreover, this knowledge is useful for the development of drugs targeting M23 peptidases that are involved in the processes essential for bacterial survival, leading to less virulent or attenuated phenotypes.

Despite multiple attractive features, the development of enzybiotics that can serve as alternatives for antibiotics poses multiple challenges. As they act rapidly, there is a risk of releasing pathogen-associated molecular patterns (PAMPs), such as PG fragments or outer membrane lipopolysaccharide (LPS), in high doses, which can potentiate undesirable and severe host immune responses (Murray et al., 2021). Their fragile, protein nature makes them difficult and expensive to produce and handle. Their stability and, consequently, their activity may be affected by multiple environmental factors during production, storage, or administration (e.g., pH, temperature, presence of other proteases, and the activity of the immune system). To summarize, much more data concerning their formulation, administration, safety, pharmacokinetics, and pharmacodynamics is needed to assess their potential use as novel antimicrobials.

Collectively, these findings highlight a new perspective in research concerning M23 peptidases and indicate that besides the prominent amount of data gathered so far, much more remains to be explored.

## Author contributions

AR: conceptualization, investigation and writing- review and editing. J-NS: investigation and writing- review and editing. PM: investigation and writing- review and editing. MK-D: conceptualization, investigation, supervision and writing – review and editing. IS: conceptualization and supervision - review and editing. All authors contributed to the article and approved the submitted version.

## Funding

The research was funded by the Foundation for Polish Science (FNP; PL) TEAMTech program (the INFECTLESS grant, a new generation of antibacterial wound dressing, POIR. 04.04.00–00–3D8D/16–00), co-financed by the Polish National Agency for Academic Exchange NAWA: International Academic Partnerships as the part of the program on the Molecular Basis of Enzyme Specificity and Applications (PPI/APM/2018/1/00034), and The National Centre for Research and Development as part of PrevEco project (NOR/POLNOR/PrevEco/0021/2019), supported by Norway grants in POLNOR2019 “Applied Research” programme.

## Conflict of interest

IS holds the patent EP2699254 “The method of proteolysis, peptidase, the composition to be used as a bacteriostatic and bactericidal agent, the mixture and the applications of the active form of LytM from *S. aureus* or derivatives thereof.”

The remaining authors declare that the research was conducted in the absence of any commercial or financial relationships that could be construed as a potential conflict of interest.

## Publisher’s note

All claims expressed in this article are solely those of the authors and do not necessarily represent those of their affiliated organizations, or those of the publisher, the editors and the reviewers. Any product that may be evaluated in this article, or claim that may be made by its manufacturer, is not guaranteed or endorsed by the publisher.

## Footnotes

^1^ https://www.staphefekt.com/

^2^ clinicaltrials.gov
